# Sex Differences in the Association between Atrial Fibrillation and 90-Day Adverse Outcomes among Older Adults with Heart Failure: A Retrospective Cohort Study

**DOI:** 10.3390/ijerph18052237

**Published:** 2021-02-24

**Authors:** Youn-Jung Son, Da-Young Kim, Mi Hwa Won

**Affiliations:** 1 Red Cross College of Nursing, Chung-Ang University, Seoul 06974, Korea; yjson@cau.ac.kr; 2 Department of Nursing, Graduate School, Chung-Ang University, Seoul 06974, Korea; dayo2316@gmail.com; 3 Department of Nursing, Wonkwang University, Iksan 54538, Korea

**Keywords:** heart failure, older adults, atrial fibrillation, sex, patient readmission, emergencies, retrospective studies

## Abstract

Sex differences in the prognostic impact of coexisting atrial fibrillation (AF) in older patients with heart failure (HF) have not been well-studied. This study, therefore, compared sex differences in the association between AF and its 90-day adverse outcomes (hospital readmissions and emergency room (ER) visits) among older adults with HF. Of the 250 older adult patients, the prevalence rates of coexisting AF between male and female HF patients were 46.0% and 31.0%, respectively. In both male and female older patients, patients with AF have a significantly higher readmission rate (male 46.0%, and female 34.3%) than those without AF (male 6.8%, and female 12.8%). However, there are no significant differences in the association between AF and ER visits in both male and female older HF patients. The multivariate logistic analysis showed that coexisting AF significantly increased the risk of 90-day hospital readmission in both male and female older patients. In addition, older age in males and longer periods of time after an HF diagnosis in females were associated with an increased risk of hospital readmission. Consequently, prospective cohort studies are needed to identify the impact of coexisting AF on short- and long-term outcomes in older adult HF patients by sex.

## 1. Introduction

Heart failure (HF) is a significant global health issue and disproportionately influences older individuals [1,2,3]. Older patients are liable to developing HF because of age-related physiologic and pathologic changes [4]. The prevalence of HF is around 11.8% in those aged 65 years and over [5]. Despite the advancement of medical therapy and technology, HF is one of the most common reasons for hospitalization and results in high morbidity, mortality, and health costs in an aging population [6,7]. The one-year global HF hospital readmission rates are from 24.3% to 30.9% [8]. Remarkably, readmission rates may be as high as 45% in older adult populations [9]. According to the Korean Acute HF registry, 446 (9.8%) of 4566 Korean patients aged ≥40 years had a 30-day HF-specific readmission [10]. One third of patients with HF use the emergency room (ER) [11]. The high proportion of patients with frequent ER visits reflects the failure of current measures to manage HF symptoms [11]. However, the proportion of readmissions and ER visits from HF that may be preventable is still unclear.

With an aging population, there is an increased likelihood of having two or more chronic diseases simultaneously [4]. It is widely perceived that comorbidities are common in HF and is associated with increased mortality, the complexity of clinical management, and poor quality of life (QOL) [12]. Particularly, atrial fibrillation (AF) which is the most common sustained arrhythmia encountered in HF [13]. The reported prevalence of AF in HF ranges from 13% to 27% [13,14]. Additionally, AF prevalence also steeply increases with age [14]. Multiple relationships can exist between HF and AF, including shared risk factors and the causation of one by the other [14,15]. Recently, two meta-analyses have reported that the coexistence of AF in HF patients increased the odds of mortality from 14% to 57% when compared to HF alone [16,17]. AF is also associated with an increased risk of readmission for HF exacerbation [18,19,20]. However, previous studies have mainly focused on the impact of AF on mortality in HF patients when compared to hospital readmission rates and ER visits as a quality of care indicator [21,22,23].

These days, sex differences in the epidemiology, comorbidity, and disease management of HF have been highlighted [24,25,26]. Although HF incidence is initially higher in men, women survive longer after the onset of the disease [24]. A recent review reported that female patients with HF were more likely to experience higher readmission rates than men when the time-span was less than a year [27]. However, the female’s contribution to the prognosis of HF has frequently been underestimated in many randomized controlled trials due to the exclusion of extreme clinical variables that are dominant in females [24,26]. As per prior studies [16,17], diabetes mellitus, hypertension, and thyroid disease are prevalent among women, whereas AF is more frequent in men. Unfortunately, sex differences in clinical characteristics and adverse outcomes in HF patients with AF have received limited attention [26,27].

Hospital readmission rates and frequent ER visits have been used as indicators for the quality of care in many hospitals [22]. Furthermore, both rehospitalization and ER visits have been associated with increased healthcare costs [13,28]. As mentioned, comorbidities and their treatments may complicate the treatment, and patient outcomes in HF, especially, older patients [15,19]. Accordingly, healthcare professionals should examine the factors that can cause adverse outcomes in older patients with HF. Evidence regarding the 90-day readmission rates are not as well established as compared to 30-day readmissions and 1-year follow-ups in Korea and other countries [27]. Particularly, the 90-day period after discharge may be more critical for observation of readmission and healthcare utilization during the vulnerable phase among the real-world Korean patients with HF [10].

Therefore, this study aimed to investigate the impact of coexisting AF on hospital readmission rates and ER visits, defined as adverse outcomes, within 90 days after hospital discharge in older patients with HF with a focus on the identification of sex differences.

## 2. Materials and Methods

### 2.1. Study Design and Sample

This was a single-center, retrospective cohort study of patients diagnosed with HF. Patients who were hospitalized initially with a primary discharge diagnosis of HF from January 2019 to December 2019 were recruited. A total 314 patients were included during the enrollment period. The date of the electronic medical record with the first HF diagnosis was defined as the index date; patients were required to be at least 65 years old on the index date. Patients with an HF diagnosis before the index date (*n* = 22) were excluded from the study. Patients lacking follow-up data after the index date (*n* = 42), such as elements to identify hospital readmission and ER visits or being transferred to another hospital including acute care hospitals, rehabilitation hospitals, or hospices, were excluded. Finally, a total of 250 patients were included in the study.

In this study, coexisting AF included pre-existing or new-onset AF. Pre-existing AF was defined as diagnosed AF occurring before an HF diagnosis. New-onset AF was defined as diagnosed AF occurring after or at the same time as an HF diagnosis. Coexisting AF was identified when it was a discharge diagnosis or was confirmed on at least two occasions in the outpatient department.

A power analysis was conducted using the G*Power 3.1 program for determining the sample size [29]. A sample size of 207 patients was required for an odds ratio of 1.9 with 95% power at the 0.05 significance level with a two-tail logistic regression analysis, based on previous results [30].

### 2.2. Ethical Considerations and Data Collection

Preceding data collection, the approval of the hospital’s Institutional Review Board was obtained. Patients’ written consent was not required since this was a retrospective study based on the guidance of the institute protocol. However, data deidentification and measures to ensure confidentiality were implemented.

A retrospective chart review using electronic medical records was performed for a consecutive series of all patients diagnosed for the first time at a tertiary acute care hospital in Gyeonggi-do, Korea.

### 2.3. Instruments

#### 2.3.1. Sociodemographic and Clinical Characteristics

Elicited from previous studies [14,31], patients’ sociodemographic data included age, sex, education level, living arrangement, and job. Clinical characteristics also covered the duration of an HF diagnosis, New York Heart Association (NYHA) functional class, left ventricular ejection fraction (LVEF), laboratory parameters including pro B type natriuretic peptide (BNP), high density lipoprotein cholesterol (HDL), low density lipoprotein cholesterol (LDL), total cholesterol, triglyceride, blood urea nitrogen, and creatinine, and other HF-related major comorbidities (hypertension, diabetes mellitus, coronary artery disease, stroke, chronic kidney disease, and dyslipidemia), and prescribed cardiac medications. All these variables were assessed during the baseline study.

#### 2.3.2. Adverse Outcomes

Data on HF-caused hospital readmissions and ER visits within 90 days after hospital discharges were compiled. Unplanned readmissions or ER visits for HF were defined as any return to the hospital within 90 days of the index admission date. The primary diagnosis documented in the patient’s chart was the reason for their return visit.

### 2.4. Statistical Analysis

The sociodemographic and clinical variables are displayed as frequencies and percentages, and the mean values as standard deviations (SD). Univariate descriptive statistics (chi-square, and independent *t*-tests) were used to identify the differences in both patient characteristics and adverse outcomes (90-day hospital readmission and ER visits) through sex and AF status. A multivariate logistic regression model was used to examine the relationship of sex differences in association with AF and the adverse outcomes after controlling for possible confounding factors. Moreover, the logistic regression analysis calculated as the odds ratio (OR) with 95% confidence interval (CI), was used to determine the appropriate fit for The Hosmer–Lemeshow test. All analyses were undertaken using the SPSS Statistics version 26 (IBM Corp., Armonk, NY, USA). A *p*-value of 0.05 or less was used.

## 3. Results

### 3.1. Patients’ Socio-Demographic and Clinical Characteristics

Of the 250 older adult patients with HF, 137 were male (54.8%), and 113 were female (45.2%). The mean age of patients was 74.44 (SD 6.07) years, and 69.2% of the patients had a below middle school education level. Majority (78.0%) of the patients were living with their families and 41 patients (16.4%) were employed. The mean duration after HF diagnosis was 7.73 (SD 4.80) years, and 49.2% of the patients were in NYHA Class I. Majority (78.8%) of the patients had an above 50% LVEF. Regarding comorbidities and prescribed cardiac medications, 112 patients had been diagnosed with hypertension and 63.2% of the patients were prescribed beta-blockers (Table 1).

Table 1 shows the prevalence of AF was 39.2% (98 patients) in older adult patients with HF. Specifically, the prevalence of AF in males (63 patients, 46%) was relatively higher than in females (35 patients, 31%). In male older adult patients with HF, there were statistically significant differences in age (χ^2^ = 7.99, *p* = 0.018), living arrangement (χ^2^ = 5.65, *p* = 0.021), taking prescribed aspirin (χ^2^ = 6.69, *p* = 0.011), and digoxin (χ^2^ = 4.47, *p* = 0.040) between patients with AF and those without AF. However, there were no significant differences in patients’ other characteristics, including education level, job, duration of HF diagnosis, NYHA class, LVEF, laboratory parameters, comorbidities, or taking prescribed cardiac medication such as warfarin, ACEI, ARB, diuretic, and beta-blockers.

In contrast, female older patients with HF had statistically significant differences in the job (χ^2^ = 5.83, *p* = 0.024), duration after an HF diagnosis (t = −2.39, *p* = 0.018), and having had a stroke (χ^2^ = 5.83, *p* = 0.024) between patients with AF and those without AF. However, there were no statistically significant differences in age, education level, living arrangement, NYHA class, LVEF, laboratory parameters, comorbidities including hypertension, diabetes mellitus, coronary artery, chronic kidney disease, and dyslipidemia, or taking prescribed cardiac medication.

### 3.2. Differences in 90-Day Hospital Readmissions and ER Visits by Gender and AF Status

The prevalence rates of the 90-day hospital readmissions and ER visits were 22.4% and 20.4%, respectively. There were statistically significant differences in the 90-day hospital readmissions by AF status for both male (χ^2^ = 28.13, *p* < 0.001) and female older adult patients with HF (χ^2^ = 7.10, *p* < 0.001). Specifically, older adult patients with HF and AF had a significantly higher 90-day hospital readmission rate (male 46.0%, and female 34.3%) than those without AF (male 6.8%, and female 12.8%) (Table 2).

However, for both older adult male and female patients with HF, there was no significant correlation between coexisting AF and their 90-day ER visits. Additionally, the 90-day ER visit rates were lower in both older adult male and female patients with HF and AF than those without AF (male 23.5%, and female, 9.8%) (Table 2).

### 3.3. Impact of Coexisting AF on 90-Day Hospital Readmissions and ER Visits by Gender

In a multivariate regression analysis, we adjusted the confounding factors that were significantly different in the univariate analyses, including patient characteristics of age, living arrangement, job, duration of HF diagnosis, stroke, aspirin, and digoxin (Table 3 and Table 4).

In the adjusted model for older adult males, coexisting AF predicted hospital readmissions within 90 days after discharge (adjusted OR 14.89, 95% CI: 14.34–45.73, *p* < 0.001). Moreover, patients within the age group of 70–79 years (adjusted OR: 8.16, 95% CI: 1.65–40.39, *p* = 0.010), and over 80 years (adjusted OR: 4.08, 95% CI: 1.05–15.88, *p* = 0.042) were also significantly associated with 90-day hospital readmissions (Table 3).

In the adjusted model for older adult females, coexisting AF also predicted hospital readmissions within 90 days after discharge (adjusted OR 6.11, 95% CI: 1.74–21.46; *p* = 0.005). Additionally, a longer period after an HF diagnosis was significantly associated with a higher risk of 90-day hospital readmission (adjusted OR 1.13, 95% CI: 1.02–1.25; *p* = 0.021) (Table 4).

However, for both older adult male and female patients with HF, coexisting AF did not predict the risk of ER visits within 90 days after hospital discharge.

## 4. Discussion

The prognostic significance of coexisting AF in patients with HF remains controversial because no consensus exists that AF is an independent risk factor of adverse outcomes [13]. In this retrospective cohort study, the overall prevalence rate of AF was 39.2% and males (46.0%) rated higher AF than females (31.0%). This finding was consistent with a prior study that reported that an incidence of AF estimated to be 1.5 to 2 times higher in men than in women after adjusting for age [32]. Similarly, Ling et al.’s [33] study determined that the prevalence rate of AF is greater in male patients than in female patients with HF. In contradiction, a prior study from Groningen, Netherlands, found that AF is an independent risk factor for new-onset HF in women but not in men [34]. A recent review reported that the differences between male and female cardiac anatomy, and electrical mechanisms, contributing to differences in risks and prognosis of AF by sex, are insufficient [35]. Compared to coronary artery disease and stroke, the differences between women and men with HF and/or AF have received less attention [36]. AF often occurs as a cause or consequence of HF [15,16]. Adverse outcomes are worse when AF and HF coexist [17,18]. In this regard, future studies are needed to identify sex-related differences in the prevalence and outcomes of these patients.

Our main finding was that in both male and female older adult patients with HF and AF, AF was a strong predictor of 90-day hospital readmissions after controlling for confounding variables. Significantly, the risk of a 90-day hospital readmission in older adult males with AF was more than twice as high as is likely for older adult females with AF. These findings were similar to a retrospective study investigating 418 older adult patients with HF that reported that the rate of hospital readmission in the AF group was significantly higher than in the normal sinus rhythm group [13]. This finding was also supported by a large study regarding hospital readmission of patients with HF, where it illustrated that AF was a significant risk factor for 90-day readmissions and that the 90-day rehospitalization rates were higher in patients with coexisting AF than in those without AF [37]. A previous study showed that the prevalence of readmission of patients with HF was significantly associated with uncontrolled AF [14]. Particularly, when AF and HF are coexisting, atrioventricular desynchrony, including impaired diastolic filling and atrial systolic dysfunction, are provoked [38]. HF and AF can exacerbate one another and lead to an increased adverse outcome [17]. Moreover, when compared to patients with HF without AF, those with AF were older and had experienced more severe symptoms [14]. Therefore, recognizing the symptoms and seriousness of AF in older adult patients with HF will help prevent negative consequences such as unplanned readmissions [13]. A recent review reported that older adult male patients with new-onset AF were at a higher risk of HF hospitalization when compared to older adult female patients without AF [39]. Our finding was also in line with previous results [39]. Namely, male HF patients with AF had a much higher risk of 90-day hospital readmission than female HF patients with AF. Customized approaches to individual prognostic factors are highly important for avoiding poor prognosis of the HF. Thus, more careful and strict management strategies should be emphasized, especially in male older patients with AF and HF.

Moreover, this study showed that older age in male patients and longer periods after an HF diagnosis in female patients were associated with an increased risk of hospital readmission. This result was supported by research that posited that an individual’s age and the duration of their illness were associated with a higher risk of readmission [17,39]. Ultimately, regarding the prevention of short-term hospital readmission, the attention of healthcare professions should be towards identifying the risk factors of individual clinical characteristics that depending on the sex. Particularly, post-discharge care or transitional care within 90 days after discharge may be a significant strategy to reduce healthcare resource utilization. Furthermore, larger cohort studies using longer follow-ups are needed to identify sex-specific risk factors to prevent adverse outcomes in older adult patients with HF.

When it comes to ER visits, more than 20% of both male and female patients with HF and without AF visited ER within 90 days after their hospital discharge. Prior research has similarly shown that frequent causes of ER visits were associated with HF exacerbation symptoms [11]. However, in the present study, coexisting AF in both male and female older adult patients with HF did not predict the risk of unplanned ER visits within 90 days after their hospital discharge. A lack of knowledge on assessing HF symptoms that require ER visits may be associated with delayed emergency care [40]. This data suggests the need for an improved understanding of the mechanisms by which different types of patients with HF are less or more likely to visit the ER as compared to being readmitted to hospital within 90 days after their discharge.

In the current study, the prevalence rate of 90-day hospital readmissions and ER visits were 22.4% and 20.4% within the total sample, respectively. This was similar to the findings of an epidemiological study that showed that 25% of older patients are readmitted within 30 days following the first hospitalization for HF [31]. In contrast, this was a lower prevalence rate than what was found in a large cohort study of adults with acute HF that established approximately 31% of patients had ER visits associated with heart issues within a one-year period after their initial discharge [11]. This discrepancy between our results and previous studies might be the reason our samples investigated patients aged 65 years and over, and the ones who have visited ER within 90 days following hospitalization. Moreover, a combination of comorbidities such as AF in older adult patients with HF can influence the rates of re-hospitalization as well as follow-up periods, and ethnic differences [35]. Therefore, health professionals should be aware of sex differences in the prognostic impact of AF on adverse outcomes. With aging populations worldwide, patients with age-related multimorbidity are becoming a norm rather than an exception. We believe that data with regard to the burden of coexisting AF among cardiovascular diseases including coronary artery diseases and peripheral artery diseases, and its impact on the short- and long-term outcomes in various subgroups, would further provide detailed insights into this high-risk patient subset.

## 5. Limitations

This study has several limitations. First, the relatively small sample size and using a Korean sample from a single-center registry means that the study’s findings cannot be generalized to larger populations. Second, the proportion of readmission cases was relatively small when compared to international data. Multicenter prospective cohort studies with a larger sample will provide more detailed information besides more precise causality. Third, this was a retrospective cohort study and suffered from inadequacy and even loss of patients’ medical records. Additionally, the influence of unknown confounding factors including European Heart Rhythm Association (EHRA) symptom classification as a symptom severity of AF [41], should not be disregarded. Some factors such as implantable cardioverter defibrillators (ICD) and cardiac resynchronization therapy (CRT) were also not included in this dataset, as those treatments might influence clinical outcomes of older patients with HF and AF. It should therefore be paid great attention to, while applying them to older patients with HF and AF. Unfortunately, there has been an underutilization of ICD and CRT in Korea [42]. These shortages are hopefully added to our future exploration. Finally, we predicted AF on adverse outcomes without considering pre-existing AF and new-onset AF. Further research is required to identify the distinct impacts of pre-existing and new-onset AF on adverse outcomes in older adult patients with HF. Moreover, our data did not address the different types of AF (paroxysmal, persistent, or permanent), which suggests that the ideal prevalence of coexisting AF may be underestimated since cases of silent paroxysmal AF may be undetected.

## 6. Conclusions

Reducing unplanned hospitalizations and frequent ER visits is an important step towards decreasing costs while maintaining high quality of care. The current study underscored the importance of sex differences on hospital readmissions in the 90 days after discharge among older adult patients with HF and coexisting AF. Our findings revealed that male patients who are more than 70 years of age were associated with higher rates of 90-day hospital readmission. In female older adult patients, a longer period after an HF diagnosis increased the risk of a hospital readmission within 90 days. Based on this data, to prevent re-hospitalizations, sex differences in older adult patients with HF and coexisting AF should be considered. This study also highlights that careful assessment and early management for coexisting AF in both male and female older adult patients with HF are vital in reducing unnecessary hospitalizations. Further research is needed to identify and characterize comorbidities and combinations of comorbidities that place HF patients at the greatest risk for adverse outcomes. Our finding will also guide future studies aimed at developing targeted HF disease management based on older patients’ comorbidity burden.

## Figures and Tables

**Table 1:** Baseline characteristics of older patients with heart failure (HF) by atrial fibrillation (AF) status.

Characteristics	All (*N* = 250)	Male (*n* = 137)				Female (*n* = 113)			
		With (*n* = 63)	Without (*n* = 74)	χ^2^/*t*	*p*	With (*n* = 35)	Without (*n* = 78)	χ^2^/*t*	*p*
	*n* (%) or M (SD)	*n* (%) or M (SD)	*n* (%) or M (SD)			*n* (%) or M (SD)	*n* (%) or M (SD)		
Age (years)	74.44 (6.07)	73.03 (6.61)	74.97 (5.24)			72.80 (6.98)	75.82 (5.59)		
65–69	61 (24.4)	22 (34.9)	12 (16.2)	7.99	0.018	13 (37.1)	14 (17.9)	5.36	0.068
70–79	135 (54.0)	27 (42.9)	48 (64.9)			14 (40.0)	46 (59.0)		
≥80	54 (21.6)	14 (22.2)	14 (18.9)			8 (22.9)	18 (23.1)		
Educational level									
Below middle school	173 (69.2)	38 (60.3)	39 (52.7)	0.80	0.393	31 (88.6)	65 (83.3)	0.52	0.578
Over high school	77 (30.8)	25 (39.7)	35 (47.3)			4 (11.4)	13 (16.7)		
Living arrangement									
Living with family	195 (78.0)	44 (69.8)	64 (86.5)	5.65	0.021	25 (71.4)	62 (79.5)	0.87	0.346
Living alone	55 (22.0)	19 (30.2)	10 (13.5)			10 (28.6)	16 (20.5)		
Job									
Yes	41 (16.4)	47 (74.6)	58 (78.4)	0.27	0.687	6 (17.1)	3 (3.9)	5.83	0.024
No	209 (83.6)	16 (25.4)	16(21.6)			29 (82.9)	75 (96.1)		
Duration of HF diagnosis (years)	7.73 (4.80)	6.59 (3.12)	8.08 (4.12)	−1.92	0.057	6.51 (3.83)	8.87 (4.32)	−2.39	0.018
NYHA class									
I	123 (49.2)	31 (49.2)	47 (63.5)	3.26	0.196	15 (42.9)	30 (38.5)	0.68	0.712
II	106 (42.4)	27 (42.9)	21 (28.4)			18 (51.4)	40 (51.3)		
III–IV	21 (8.4)	5 (7.9)	6 (8.1)			2 (5.7)	8 (10.2)		
LVEF (%)									
≤40	27 (10.8)	10 (15.9)	9 (12.2)	3.92	0.141	3 (8.6)	5 (6.4)	0.22	0.894
41–49	26 (10.4)	3 (4.8)	11 (14.9)			4 (11.4)	8 (10.3)		
≥50	197 (78.8)	50 (79.3)	54 (72.9)			28 (80.0)	65 (83.3)		
Laboratory parameters									
pro BNP (pg/mL)	223.20 (558.85)	67.80 (54.03)	147.06 (104.87)	−1.46	0.147	253.91 (189.29)	404.72 (954.49)	−0.41	0.684
HDL (mg/dL)	51.08 (31.57)	49.88 (14.30)	49.45 (16.19)	0.12	0.902	50.50 (16.21)	53.56 (50.07)	−0.28	0.781
LDL (mg/dL)	82.71 (30.19)	74.88 (27.55)	86.00 (30.00)	−1.66	0.101	83.06 (31.48)	85.11 (31.62)	−0.23	0.816
Total cholesterol (mg/dL)	158.59 (51.56)	150.92 (34.31)	167.53 (80.12)	−1.35	0.181	163.54 (34.94)	154.71 (32.88)	1.14	0.259
Triglyceride (mg/dL)	146.49 (127.11)	139.61 (118.71)	164.13 (169.96)	−0.85	0.400	130.77 (68.24)	142.75 (107.68)	−0.53	0.600
Blood urea nitrogen (mg/dL)	18.83 (10.49)	17.62 (6.49)	19.67 (9.09)	−1.46	0.147	17.13 (6.91)	19.85 (14.81)	−1.01	0.316
Creatinine (mg/dL)	1.12 (0.50)	67.80 (54.03)	1.12 (0.38)	−0.23	0.817	1.19 (1.03)	1.10 (0.37)	0.66	0.511
Comorbidities									
Hypertension, yes	112 (44.8)	22 (34.9)	29 (39.2)	0.27	0.723	17 (48.6)	44 (56.4)	0.59	0.541
Diabetes mellitus, yes	80 (32.0)	20 (31.7)	17 (22.9)	1.33	0.334	12 (34.3)	31 (39.7)	0.31	0.677
Coronary artery disease, yes	60 (24.0)	14 (22.2)	19 (25.7)	0.22	0.692	6 (17.1)	21 (26.9)	1.27	0.342
Stroke, yes	20 (8.0)	7 (11.1)	4 (5.4)	1.50	0.345	6 (17.1)	3 (3.8)	5.83	0.024
Chronic kidney disease, yes	11 (4.4)	4 (6.3)	3 (4.1)	0.37	0.703	1 (2.9)	3 (3.8)	0.61	0.664
Dyslipidemia, yes	15 (6.0)	3 (4.8)	7 (9.5)	1.11	0.342	2 (5.7)	3 (3.8)	3.76	0.087
Prescribed cardiac medications									
Aspirin, yes	113 (45.2)	21 (33.3)	41 (55.4)	6.69	0.011	12 (34.3)	39 (50.0)	2.41	0.153
Warfarin, yes	66 (26.4)	19 (30.2)	19 (25.7)	0.34	0.572	23 (65.7)	62 (79.5)	2.46	0.157
ACEI, yes	32 (12.8)	6 (9.5)	15 (20.3)	3.03	0.099	1 (2.9)	10 (28.6)	2.73	0.168
ARB, yes	87 (34.8)	22 (34.9)	23 (31.1)	0.23	0.716	14 (40.0)	28 (35.9)	0.17	0.680
Diuretic, yes	107 (42.8)	30 (47.6)	27 (36.5)	1.74	0.225	19 (54.3)	31 (39.7)	2.07	0.159
Digoxin, yes	72 (38.8)	24 (38.1)	16 (21.6)	4.47	0.040	14 (40.0)	18 (23.1)	3.41	0.075
Beta blocker, yes	158 (63.2)	42 (66.7)	45 (60.8)	0.50	0.593	13 (37.1)	29 (37.2)	0.01	0.997

HF: Heart failure; AF: atrial fibrillation; BNP: B type natriuretic peptide; HDL: high-density lipoprotein cholesterol; LDL: low-density lipoprotein cholesterol; ACEI: angiotensin-converting enzyme inhibitor; ARB: angiotensin II receptor blocker; LVEF: left ventricular ejection fraction; NYHA: New York Heart Association.

**Table 2:** 90-day hospital readmissions and emergency room (ER) visits by AF status in older patients with HF.

Characteristics	All (*N* = 250)	Male (*n* = 137)				Female (*n* = 113)			
		With (*n* = 63)	Without (*n* = 74)	χ^2^/*t*	*p*	With (*n* = 35)	Without (*n* = 78)	χ^2^/*t*	*p*
	*n* (%) or M (SD)	*n* (%) or M (SD)	*n* (%) or M (SD)			*n* (%) or M (SD)	*n* (%) or M (SD)		
90-day hospital readmissions									
No	194 (77.6)	34 (54.0)	69 (93.2)	28.13	<0.001	23 (65.7)	68 (87.2)	7.10	0.011
Yes	56 (22.4)	29 (46.0)	5 (6.8)			12 (34.3)	10 (12.8)		
90-day ER visits									
No	199 (79.6)	51 (81.0)	58 (78.4)	0.14	0.832	30 (85.7)	60 (76.9)	1.15	0.324
Yes	51(20.4)	12 (19.0)	16 (21.6)			5 (14.3)	18 (23.1)		

HF: Heart failure; AF: atrial fibrillation; ER: Emergency room.

**Table 3:** Multivariate logistic regression analysis of 90-day hospital readmissions and ER visits in older male patients with HF (*n* = 137).

Variables (Reference)	90-Day Hospital Readmissions			90-Day ER Visits		
	Adjusted OR	95% CI	*p*	Adjusted OR	95% CI	*p*
Age (65–69 years)						
70–79	8.16	1.654–0.39	0.010	3.88	0.98–15.33	0.053
≥80	4.08	1.05–15.88	0.042	1.57	0.29–8.39	0.596
Living arrangement (Living with family)						
Living alone	3.12	0.95–10.21	0.060	1.18	0.41–3.42	0.760
Job (No)						
Yes	1.70	0.46–6.32	0.429	1.24	0.39–3.90	0.709
Duration of HF diagnosis (years)	0.94	0.80–1.09	0.421	1.01	0.91–1.11	0.919
Stroke (No)						
Yes	0.42	0.06–2.75	0.365	0.39	0.05–3.36	0.390
Aspirin (No)						
Yes	0.63	0.27–2.22	0.633	0.99	0.41–2.41	0.980
Digoxin (No)						
Yes	1.12	0.36–3.45	0.849	0.76	0.27–2.13	0.606
Presence of AF (No)						
Yes	14.89	4.34–45.73	<0.001	1.18	0.45–3.08	0.739

HF: heart failure; AF: atrial fibrillation; OR: odds ratio; CI: confidence interval; ER: emergency room.

**Table 4:** Multivariate logistic regression analysis of 90-day hospital readmissions and ER visits in older female patients with HF (*n* = 113).

Variables (Reference)	90-Day Hospital Readmissions			90-Day ER Visits		
	Adjusted OR	95% CI	*p*	Adjusted OR	95% CI	*p*
Age (65–69 years)						
70–79	1.73	0.38–7.96	0.511	0.48	0.14–1.70	0.257
≥80	1.74	0.33–9.06	0.476	0.93	0.24–3.57	0.910
Living arrangement (Living with family)						
Living alone	2.36	0.69–7.96	0.168	0.97	0.31–3.06	0.956
Job (No)						
Yes	4.35	0.38–50.49	0.239	2.21	0.19–25.75	0.527
Duration of HF diagnosis (years)	1.13	1.02–1.25	0.021	1.05	0.96–1.15	0.273
Stroke (No)						
Yes	4.12	0.78–21.88	0.096	1.78	0.29–10.99	0.536
Aspirin (No)						
Yes	1.75	0.58–5.27	0.318	1.11	0.43–2.92	0.828
Digoxin (No)						
Yes	1.02	0.322–3.23	0.974	0.98	0.33–2.85	0.965
Presence of AF (No)						
Yes	6.11	1.74–21.46	0.005	0.55	0.16–1.87	0.337

HF: heart failure; AF: atrial fibrillation; OR: odds ratio; CI: confidence interval; ACEI: angiotensin-converting enzyme inhibitor; ER: emergency room.

## Data Availability

Data sharing is not applicable to this article as data analyzed in the current study contains participant medical information. Sharing of this information would breach patient privacy, confidentiality and the ethics approval gained for the study.
